# Shoot Tip Cryopreservation of *Lamprocapnos spectabilis* (L.) Fukuhara Using Different Approaches and Evaluation of Stability on the Molecular, Biochemical, and Plant Architecture Levels

**DOI:** 10.3390/ijms21113901

**Published:** 2020-05-30

**Authors:** Dariusz Kulus

**Affiliations:** Laboratory of Ornamental Plants and Vegetable Crops, Faculty of Agriculture and Biotechnology, UTP University of Science and Technology in Bydgoszcz, Bernardyńska 6, 85-029 Bydgoszcz, Poland; dkulus@gmail.com

**Keywords:** biochemical array, genetic stability, ISSR, medicinal plants, molecular markers, long-term storage, PCR, PVS3, RAPD, SCoT

## Abstract

The aim of this study is to optimize and evaluate the effectiveness of vitrification, droplet-vitrification, and encapsulation-vitrification techniques in the cryopreservation of *Lamprocapnos spectabilis* (L.) Fukuhara ‘Gold Heart’, a popular medicinal and ornamental plant species. In vitro-derived shoot tips were used in the experiments. All three techniques were based on explant dehydration with plant vitrification solution 3 (PVS3; 50% glycerol and 50% sucrose) for 0, 30, 60, 90, 120, 150, or 180 min. The recovered microshoots were subjected to morphometric, biochemical, and molecular analyses (RAPD, ISSR, SCoT). The highest recovery level was reported with the encapsulation-vitrification protocol based on 150 min dehydration (73.1%), while the vitrification technique was the least effective (maximum 25.8% recovery). Explants cryopreserved with the encapsulation-vitrification technique produced the highest mean number of shoots (4.9); moreover, this technique was optimal in terms of rooting efficiency. The highest fresh weight of shoots, on the other hand, was found with the vitrification protocol based on a 30-min PVS3 treatment. The concentrations of chlorophyll *a* and *b* were lower in all cryopreservation-derived plants, compared to the untreated control. On the other hand, short dehydration and cryopreservation of non-encapsulated explants stimulated the synthesis of anthocyanins. A small genetic variation in 5% of all samples analyzed was detected by RAPD and ISSR marker systems. Only plants recovered from the encapsulation-vitrification protocol had no DNA sequence alternations.

## 1. Introduction

*Lamprocapnos spectabilis* (L.) Fukuhara (bleeding heart) is a popular ornamental plant species. It is commonly grown in parks and gardens in North America, Europe, and Asia [1,2]. The species has huge potential in the pharmaceutical and cosmetics industries [3,4,5], being a source of natural fungitoxic alkaloids [6]. Unfortunately, despite its popularity, the number of endemic populations of the species is limited and it may be placed into the endangered category soon [2]. There is also limited information on in vitro tissue culture technology with *L. spectabilis* [7,8,9]. Conservation of the genetic resources of species is an important task to secure valuable genotypes and cell lines (e.g., for breeding and sustainable development). At present, cryopreservation—the storage of biological material in liquid nitrogen (LN, −196 °C) or liquid nitrogen vapor (LNV, −165 to −196 °C)—is considered to be the most successful long-term storage method for plant diversity [10].

Most available cryopreservation techniques are based on tissue dehydration and transition of the remaining bound water into an amorphous biological glass state (i.e., vitrification). By such means, lethal ice crystallization during sub-zero temperature storage and consequent cell-bursting can be avoided. Vitrification can be achieved in several ways, such as slow or ultra-fast cooling. Slow cooling is no longer common, as it requires expensive equipment. The so-called modern cryopreservation techniques by ultra-fast cooling are much more popular and efficient [10]. In this approach, after an earlier preculture, explants are usually pretreated with a plant vitrification solution (PVS), which consists of a mixture of cryoprotectants—that is, protective agents which dehydrate the cell, stabilize cell membranes, increase the viscosity of the cytosol, and lower the freezing point—before storage in LN. The first PVS (PVS2) was optimized by Sakai et al. [11] and consisted of 30% glycerol (*w/v*), 15% ethylene glycol (*w/v*), 15% dimethyl sulfoxide (*w/v*; Me_2_SO), and 0.4 M sucrose. Me_2_SO, however, may be toxic, causing changes in the permeability of plasmalemma, enzyme activity, and oxygen uptake. Moreover, it affects the structure of chromosomes and the expression of genes [12]. For these reasons, Nishizawa et al. [13] developed PVS3 (50% glycerol *w/v* and 50% sucrose *w/v*), which is preferable for explants which are damaged by Me_2_SO. One should keep in mind, though, that not only the type and concentration of PVS but also the exposure duration and the temperature of cryoprotectant should be carefully optimized to achieve a high regrowth level of the cryopreserved biological material [14].

To improve the effectiveness of cryopreservation and increase the chances of explant recovery without genetic perturbation (besides PVS treatment), explants can be additionally encapsulated in calcium alginate beads, such as in the encapsulation-vitrification technique originally developed by Matsumoto et al. [15], or placed in a drop of PVS on aluminum foil strip, which accelerates the cooling and rewarming rates (the so-called droplet-vitrification technique developed by Panis et al. [16]). The choice of optimal cryopreservation technique is often species dependent [17]. To date, however, there has been no research on cryo-storage of the *Lamprocapnos* genus or any other member of its botanical family, Fumariaceae.

Stability and uniformity of the recovered plant after cryo-exposure is a major concern in the cryopreservation of plant genetic resources. No or minor variation is often detected after tissue storage in LN [18]. However, Martín et al. [19] reported significant genetic variation after applying the encapsulation-dehydration cryopreservation protocol (based on osmotic dehydration of tissues in sucrose gradient) in *Chrysanthemum × morifolium* Ramat. Pawłowska and Szewczyk-Taranek [20] observed the influence of cryopreservation by droplet-vitrification on the morphogenetic events in members of the *Rosa* genus. Moreover, Hitmi et al. [21] found that the capability to synthesize pyrethrin was significantly improved in cryopreserved cell lines of *Chrysanthemum cinerariaefolium* Vis. On the other hand, no change in bacoside content was reported in *Bacopa monnieri* (L.) Wettst regenerated from shoot tips cryopreserved by the vitrification technique [22]. Biochemical stability is particularly important for plants used for medicinal purposes; it directly affects the physiological quality of plants and vigor [23]. Unfortunately, there has been limited research comparing the effects of different cryopreservation protocols on genetic and biochemical stability in a single plant species [24].

The aim of this study was to optimize and evaluate, for the first time, the effectiveness of three cryopreservation techniques—vitrification, droplet-vitrification, and encapsulation-vitrification—in terms of regrowth of *L. spectabilis* shoot tips and their morphogenetic response in post-rewarming culture. The genetic and biochemical stability of in vitro-recovered plantlets was also compared after applying the various cryo-procedures.

## 2. Results

### 2.1. Recovery and Morphogenetic Potential of Shoot Tips Cryopreserved by Vitrification, Droplet-Vitrification, and Encapsulation-Vitrification Techniques 

The highest recovery rate (100%) was reported for non-encapsulated, untreated shoot tips and after LS treatment for 20 min (Table 1). All other treatments had a negative impact on this parameter. The viability of the “naked” explants declined with the duration of dehydration, regardless of LN storage. Encapsulated shoot tips, on the other hand, showed lower variability in the recovery response.

The droplet-vitrification and encapsulation-vitrification techniques were more effective in securing the shoot tips of bleeding heart ‘Gold Heart’ (22.8–52.4% and 32.4–73.1% recovery level, respectively), compared to the vitrification technique (6.4–25.8%). The highest recovery level (73.1%) of cryopreserved samples was found with the encapsulation-vitrification technique based on 150 min PVS3 exposure duration (Table 1). None of the control shoot tips or those osmoprotected only with LS (0 min PVS3) or those encapsulated and dehydrated with PVS3 for 30 min survived storage in LN. Therefore, they were not included in further analyses.

The cryopreservation procedure affected the morphogenetic response of the shoot tips (see Table 1). Explants cryopreserved with encapsulation-vitrification and 120 min PVS3 treatment produced a higher mean number of shoots (4.9) than most other treatments, except for EV150 (3.9 shoots). The plant proliferation ratios in other experimental combinations were similar (1.0–3.1). 

All plants recovered from the droplet-vitrification technique and most plants from the vitrification protocols (90–180 min PVS3 exposure) were significantly shorter (5.8–14.0 mm) than the longest shoots grown from encapsulated and LS-treated explants (non-cryopreserved), and after using the vitrification technique and 30 min dehydration (approx. 29 mm; Table 1). As for the encapsulation-vitrification technique, only one experimental combination (with 60 min dehydration) produced shoots shorter (14.0 mm) than the longest shoots in the other treatments.

Shoots recovered from the vitrification technique based on 30 min dehydration had the highest fresh weight (2412.5 mg), compared to all other treatments (Table 1). Furthermore, plants after encapsulation-vitrification and 120 min PVS3 exposure had a higher FW (951.8 mg) than those from most other experimental combinations, including the control (198.3 mg).

No rhizogenesis was reported in the untreated control (Table 1). In contrast, explants from the four experimental combinations (i.e., non-encapsulated and PVS3-treated for 30 min, encapsulated but untreated or osmoprotected only with LS, and those from the EV cryopreservation technique based on 120 min dehydration) regenerated significantly more roots (31.2–41.2% rooting efficiency). Shoots recovered after cryopreservation by encapsulation-vitrification regenerated roots more often (25.6% mean, regardless of dehydration duration) than those from the vitrification and droplet-vitrification methods (1.7–3.5%).

### 2.2. Biochemical Evaluation of Recovered Shoots

The highest content of chlorophylls in the shoots of explants was found in the untreated control (chlorophyll *a*, *b*, *ct*) and after 30–60 min PVS3 treatments (chlorophyll *b* and *ct*; Table 2). All other treatments (with or without LN storage) had a deleterious effect on those parameters. For example, the chlorophyll *a* and *b* concentrations in the untreated control accounted for 3.24 and 1.21 mg per gram of fresh matter while, after the encapsulation-vitrification cryopreservation with 180 min PVS treatment, it decreased to 0.47 and 0.17 mg, respectively. There were no differences in the content of chlorophylls in the LN-derived shoots, regardless of the cryo-protocol applied.

The content of anthocyanins in the untreated control was 5.38 mg·g^−1^ FW. A significantly higher concentration (9.01–10.73 mg·g^−1^) was found in LN-derived shoots under the vitrification and droplet-vitrification techniques based on 30 min dehydration (Table 2). The content of anthocyanins was stable in bleeding hearts produced from encapsulated non-LN-stored explants, regardless of dehydration duration (3.59–6.76 mg·g^−1^). In contrast, in all three cryopreservation techniques studied, the longest PVS3 treatments (with vitrification also 150 min) diminished the concentration of those secondary metabolites, compared to the untreated control (0.51–1.65 mg·g^−1^). 

Plants recovered after cryopreservation by vitrification with 150- and 180-min PVS3 treatments had a significantly higher chlorophyll *ct* to anthocyanins ratio (1.92–2.18), compared to all other experimental combinations (0.11–0.82; Table 2).

### 2.3. Molecular Evaluation of LN-Recovered Shoots

**Figure 1:**
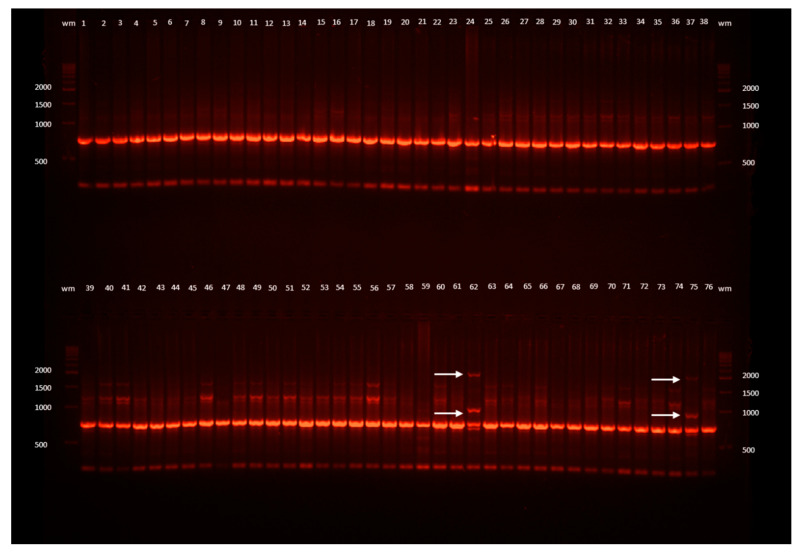
Example ISSR band profiles of *L. spectabilis* ‘Gold Heart’ received as a result of electrophoresis of the DNA amplification products obtained with the I-D primer. Outermost lanes (wm) are DNA bp weight markers; 1, 2, 3, ... are the numbers of the plants: 1–29 recovered after encapsulation-vitrification (EV) cryopreservation, 30–54 after droplet-vitrification (DV), 55–70 after vitrification (V), 71–76 untreated control plants (C); arrows point to changed band profiles.

The mean numbers of markers generated by a single RAPD, ISSR, and SCoT primer were as follows: 7.2, 6.7, and 8.5, respectively (Table 3). The size of bands ranged from 36 bp to 3158 bp. Three ISSR primers (I-C, I-E, and I-F) failed to generated unambiguous amplicons; therefore, they were not included in the study. Among the molecular marker systems tested, ISSRs generated most polymorphisms: a mean of 37.0% polymorphic markers in three samples (i.e., 3.8% of all plants analyzed). Primer I-A generated the highest number of bands (eight per sample); it was also the most effective in screening for variation (62.5% polymorphic markers in two plants). On the other hand, primer I-B produced only five bands (among which, 20% were polymorphic in one sample). Specific markers were the least numerous (0.3 per primer), followed by polymorphic (2.3) and monomorphic ones (4.0). As for RAPD analysis, only primer R-F detected one polymorphic and one specific marker in a single plant (1.2% of all plants tested). No polymorphisms were detected by SCoTs, despite this marker system producing the highest mean number of markers per primer (8.5), with primer S-B generating the highest number of bands (15 per sample) and primer S-A generating the lowest number (5). An example visualization of PCR products is shown in Figure 1.

Jaccard’s coefficient of similarity value varied from 0.65–1.0, indicating low genetic diversity in the samples studied. Following the UPGMA analysis based on the RAPD results, two clusters could be distinguished. The first one was formed only by genotype V150 (cryopreserved by the vitrification technique and 150 min dehydration) with 95.3% genetic similarity to the other samples. The remaining monomorphic individuals (with 100% similarity) were grouped in the second cluster. As for the ISSR analysis, two clusters were also found. Genotypes V90 (vitrification, 90 min PVS3 treatment) and C5 (untreated control no. 5) were placed into the first cluster, with linkage similarity level of 65%. The second cluster was divided into two sub-clusters: Genotype DV120 (droplet-vitrification, 120 min dehydration) was distinguished at the level of 95% similarity and placed in the first sub-cluster. The remaining 77 monomorphic individuals were grouped into the other sub-cluster.

The AMOVA analysis revealed that 98% of the total genetic variation detected with the RAPD marker system was of inter-individual origin. As for the ISSR markers, cryo-treatment was the source of 3% of genetic variation among the four population groups tested.

## 3. Discussion

### 3.1. Survival and Recovery of Shoot Tips Cryopreserved by Vitrification, Droplet-Vitrification, and Encapsulation-Vitrification 

The survival of plant material at the temperature of liquid nitrogen is possible only after its proper pretreatment and optimization of the cryopreservation procedure. Explant dehydration is the most important step [17]. In the present study, it was found that shorter PVS3 treatment duration is more effective with vitrification (60 min) and droplet-vitrification techniques (30–60 min), in terms of explant recovery potential, while longer dehydration (150 min) duration is recommended with encapsulation-vitrification; this could be due to the presence of the bead matrix. On one hand, shoot tips in encapsulation-based cryopreservation techniques have additional physical protection; however, at the same time, they require a longer period of PVS diffusion time. This could also explain the nil survival of shoot tips dehydrated for 30 min in the EV technique. As for non-encapsulated explants, longer PVS3 treatments resulted in survival decline, regardless of LN storage. This is probably a result of osmotic shock and increasing activity of free radicals, which has been described also in droplet-vitrification cryopreserved *Solanum lycopersicum* L. shoot tips [25].

Among the three cryopreservation techniques studied, the encapsulation-vitrification protocol had several distinct advantages over vitrification and droplet-vitrification. The survival level of shoot tips cryopreserved by encapsulation-vitrification was higher under optimal PVS3 conditions, and the range of PVS3 exposure durations, which provided satisfactory survival (over 40%) was broader (90–180 min). Moreover, the handling of explants was easier, such that the entire procedure is more user friendly, although it was more time consuming due to the additional encapsulation step. In the present study, vitrification was the least effective in securing the viability of *L. spectabilis* shoot tips (6.4–25.8%). As for *Chrysanthemum morifolium* ‘Escort’, vitrification was the most successful technique, compared to slow cooling, encapsulation-dehydration, and droplet-vitrification methods [26]. The droplet-vitrification technique, on the other hand, has been successfully applied to members of the Musaceae family [16]. This highlights the issue of species dependency when choosing a cryo-protocol.

Not all plant species/materials are equally suitable for LN storage [10,17]. The recovery levels observed with *L. spectabilis* were quite high (even up to 73.1%). In comparison, with four chrysanthemum cultivars, a similar 55.3–80.3% regrowth from shoot tips was reported, but only after using a more complex encapsulation-vitrification dehydration protocol (based on 180 min LS treatment, followed by 60 min PVS3 dehydration and 4 h desiccation) [27]. As for *Ajania pacifica* (Nakai) Bremer et Humphries ‘Bengo’, a maximum of 8.3% cryopreservation-derived shoot tips produced shoots with the encapsulation-dehydration technique [28]. The survival frequency of LN-stored protocorm-like bodies (PLBs) of *Vanda coerulea* Griff. ex Lindl. was 5% [29]. Therefore, bleeding heart is a suitable model species for cryopreservation studies and can be easily stored in cryogenic banks.

### 3.2. Morphogenetic Response of Explants 

The morphogenetic response of bleeding heart explants depended on the protocol applied and, interestingly, also on storage in LN. More shoots (4.9 per viable explant) were produced with the optimized encapsulation-vitrification technique, compared to vitrification and droplet-vitrification. However, the positive impact of encapsulation on this parameter was not confirmed with non-LN-stored plant materials. In fact, explants encapsulated and dehydrated for 120–150 min produced fewer shoots (1.3–1.4) than the corresponding cryopreserved treatments (3.9–4.9). This may suggest a significant impact of low-temperature stress on shoot proliferation. Transmission electron microscopy (TEM) observations of *Chrysanthemum × grandiflorum* (Ramat.) Kitam. shoot tips revealed that cryoinjury of the apical meristem may lead to the activation of axillary buds and development of multiple shoots [30], which could also explain the present results. 

Plants produced in the presence of hydrogel matrix usually had a similar length (15.1–17.9 mm) to the longest shoots (approx. 29 mm), which was not the case in all droplet-vitrification- and most vitrification-derived shoots (5.8–14.0 mm). This was probably due to better access to nutrients present in the artificial endosperm. Moreover, dehydration is more balanced in the presence of a bead matrix and less “stressful” to the explant [28]. On the other hand, the vitrification technique was superior, in terms of shoot fresh weight. This is possibly due to easier gas exchange in non-encapsulated explants. Another explanation is the hyperhydricity of shoots reported sometimes with this technique, resulting from extensive and abrupt dehydration–rehydration cycles [17].

In the present study, it was found that none of the untreated control explants regenerated roots. However, encapsulation alone or with subsequent LS treatment positively affected the rhizogenesis efficiency in non-LN-stored shoot tips (33.7–35.0%). A high rooting level was reported also after explant cryopreservation by encapsulation-vitrification with 120 min PVS3 treatment (41.2%). No influence of encapsulation or dehydration on rooting efficiency was reported with *Ajania pacifica* ‘Bengo’, although encapsulation positively affected the fresh weight and length of roots [28]. On the other hand, encapsulation had a negative impact on the in vitro rooting of several *Chrysanthemum × grandiflorum* cultivars [31]. This suggests a significant impact of genetic factors on this trait. Perhaps the addition of auxins or gibberellic acid (GA_3_) to the artificial endosperm could improve rooting in bleeding heart, as reported with synthetic seeds of *Salvia officinalis* L. [32].

### 3.3. Stability of Plant Material after Various (Cryo)Treatments 

It is generally assumed that metabolic activity, cell division, and aging processes are nearly arrested at the temperature of LN. However, various treatments pre- and post-LN storage may affect plant metabolism [33]. Encapsulation alone had a negative impact on the concentration of chlorophylls *a* and *b* in bleeding heart plants. Furthermore, dehydration (in most cases) and cryopreservation (regardless of the protocol applied) adversely affected the content of chlorophylls in the recovered shoots. Similar results were reported by Zevallos et al. [23] with *Solanum lycopersicum* plants produced from seeds directly plunged in LN. Chlorophyll is the most labile pigment within the plant. During stress, inhibition in the activity of enzymes related to chlorophyll biosynthesis, such as δ-aminolevulinic acid dehydratase and protochlorophyllide reductase, has been observed [34]. This may explain the decline in content observed here. Interestingly, in the present study, there were no differences in the concentration of chlorophylls in plants recovered from the various cryo-protocols. This lack of variation suggests that explants which survived storage in LN were similarly well-preserved against the activity of free radicals produced under stress conditions [23,25].

Although they are valuable medicinally, due to anticancer, antioxidant, and other health-promoting properties [35], not enough attention has been focused on the synthesis of anthocyanins after cryopreservation [36,37]. In the present study, the short dehydration and cryopreservation of “naked” explants stimulated the production of these metabolites, as their concentration after 30 min PVS3 exposure was nearly twofold higher (9.01–10.73 mg·g^−1^ FW), compared to the untreated control (5.38 mg·g^−1^). This change can be considered positive for the horticultural and pharmaceutical industries. An increase in anthocyanins content in cells is a natural reaction to stress, which has also been reported in other plant species [38]. Surprisingly, this phenomenon was not observed with longer dehydration durations. The content of anthocyanins even decreased with the longest PVS3 treatment in all three cryopreservation techniques studied (and with 150 min in vitrification). Similarly, the levels of cell wall-linked, free, and total phenolics declined in roots and stems of cryopreservation-recovered *Solanum lycopersicum* plants, compared to non-cryopreserved control [33]. This may suggest a deleterious influence of severe stress on the native structure of anthocyanins [34] and explain the drastic increase in total chlorophyll to anthocyanins ratio in the less-effective cryopreservation protocols (i.e., V150 and V180). Alternatively, stress-related microfractures in the cell compartment might have put together some biochemicals which are normally isolated, inducing alterations in different metabolic pathways [33]. According to Shibli et al. [36], LN-derived cells regain their maximum capacity for pigment accumulation after 1–3 subcultures, which may also be the case with *L. spectabilis*.

Martín et al. [19] suggested that each step of a cryopreservation protocol may be a source of genetic variation. According to the authors, such variation may be associated with tissue culture conditions (e.g., non-optimized media), and not necessarily with LN damage. The results obtained in this study indicate a low level of genetic variability within just four plants tested (5% of all analyzed samples). Interestingly, the same seven polymorphic markers detected in one LN-derived sample (revealed by two ISSR primers) were found also in one untreated control plant. This may indicate the presence of some hotspots with a high frequency of mutation occurrence in the genome of bleeding heart [39]. A similarly low genomic variation—at the level of 5%—was reported in LN-stored *Hladnikia pastinacifolia* Rchb. [18] and *Chrysanthemum × grandiflorum* [36]. The results obtained here suggest that, with no altered genotypes detected, the encapsulation-vitrification technique not only secures the viability of the highest number of explants, but is also effective in preserving the genetic stability of plants. On the other hand, the droplet-vitrification and vitrification techniques may be a source of some minor variation, probably due to a lack of additional physical protection of the explant.

## 4. Materials and Methods 

### 4.1. Plant Material and Its Multiplication

*Lamprocapnos spectabilis* (L.) Fukuhara ‘Gold Heart’ was selected for cryopreservation protocol optimization. In vitro cultures of bleeding heart were maintained in an actively growing state on modified Murashige and Skoog (MS) medium [40] supplemented with extra 330 mg·L^−1^ calcium II chloride (CaCl_2_·6H_2_O), 13.9 mg·L^−1^ iron sulfate (FeSO_4_), 55.8 mg∙L^−1^ Na_2_EDTA·2H_2_O, 2.22 μM (0.5 mg·L^−1^) 6-benzyladenine (BA), 0.09M (30 g·L^−1^) sucrose, and 8 g·L^−1^ agar (Biocorp, Warsaw, Poland). For all experiments, the pH of culture media was adjusted to 5.8 using either HCl or NaOH (Chempur, Piekary Śląskie, Poland) before autoclaving the medium for 20 min at 105 kPa and 121 °C. Cultures were kept in a growth room at 23 °C ± 1 °C, under a photoperiod of 16 h light with a photosynthetic photon flux density (PPFD) of approximately 29.4 µmol·m^−2^·s^−1^ provided by standard cool daylight TLD 54/36 W fluorescent tubes with a color temperature of 6200 K (Koninklijke Philips Electronics N.V., Eindhoven, the Netherlands). The duration of the in vitro multiplication subculture was set as 16 weeks.

### 4.2. Preculture

Single nodes (1 cm long without leaves) were cut from a 16-week-old stock plant and placed in 350 glass jars with 30 mL of modified MS medium containing 0.27 M (90 g·L^−1^) sucrose, 4.65 μM (1.0 mg·L^−1^) kinetin, 10 µM (2.62 mg·L^−1^) of abscisic acid, and 8.0 g·L^−1^ agar at a density of 10 single nodes per jar. Plant growth regulators were provided by Sigma-Aldrich^®^, St. Louis, MO, USA. Single nodes were cultured at the same conditions as stock cultures for one week to provide shoot tips for cryopreservation experiments.

### 4.3. Cryopreservation

Shoot tips (1.0–2.0 mm in length) containing 2–3 young leaf primordia were used for cryo-processing by vitrification (V), droplet-vitrification (DV), and encapsulation-vitrification (EV) techniques. 

#### 4.3.1. Vitrification 

Shoot tips were osmoprotected with loading solution (LS; 2.0 M glycerol and 0.4 M sucrose provided by Chemia, Bydgoszcz, Poland) for 20 min at room temperature. The explants were then dehydrated in PVS3 (50% glycerol *w/v* and 50% sucrose *w/v*) for 0, 30, 60, 90, 120, 150, or 180 min at room temperature. The PVS3-treated shoot tips were transferred to 2.0 mL sterile polypropylene cryovials (10 explants per vial) containing 0.5 mL of PVS3 and plunged into LN. The control comprised of osmoprotected shoot tips treated with PVS3 (for 0–180 min) at room temperature, but not immersed in LN.

#### 4.3.2. Droplet-Vitrification 

The explants were pretreated in LS (20 min at room temperature) and PVS3 for 0, 30, 60, 90, 120, 150, or 180 min at room temperature. The PVS3-treated shoot tips were placed into drops of PVS3 on sterile aluminum foil strip (three explants per aluminum foil strip) and then plunged into LN. After LN exposure, the foils with shoot tips were transferred into a 2.0 mL sterile polypropylene cryovial (two foil strips per vial) and stored in LN. 

#### 4.3.3. Encapsulation-Vitrification 

Shoot tips were embedded in liquid modified MS medium salts, without calcium and supplemented with 3.0 g·L^−1^ sodium-alginate (Carlo Erba, Val-de-Reuil, France) and 0.27 M sucrose, for 10 min at room temperature. Then, shoot tips were individually collected and dropped into a 0.1-M CaCl_2_ solution for 30 min at room temperature. Encapsulated explants (3–4 mm in diameter) were cultured in LS (20 min at room temperature) and then with PVS3 for 0, 30, 60, 90, 120, 150, or 180 min at room temperature. Ten beads covered with PVS3 were transferred into a 2.0-mL sterile polypropylene cryovial and immersed in LN. The control comprised of encapsulated and osmoprotected shoot tips treated with PVS3 (for 0–180 min) at room temperature, but not immersed in LN.

### 4.4. Rewarming and Recovery 

After at least one hour of storage, the cryovials were removed from LN and rewarmed rapidly in a water bath (39 ± 1 °C) for 3 min. The PVS3 was removed from the vials and the explants were rinsed with washing solution (WS; liquid MS medium with 1.2 M sucrose) for 30 min. WS was replaced after half-time exposure. Thereafter, the shoot tips were inoculated onto a modified MS recovery medium supplemented with 0.09 M sucrose, 2.22 μM (0.5 mg·L^−1^) BA, and 8.0 g·L^−1^ agar. The cultures were kept for 48 h at 23 ± 1 °C in darkness and then transferred to a 16-h photoperiod and kept at a light intensity of approximately 12.8 µmol·m^−2^·s^−1^ for 5 days. Then, the cultures were grown in the same conditions as the in vitro stock plants.

### 4.5. Survival and Morphometric Analyses 

The recovery level [%] of shoot tips (i.e., their capability to form shoots) was measured 60 days after rewarming. The total number of dissected shoot tips was considered 100%. The number, length [mm], and fresh weight (FW) [mg] of shoots produced after 60 days of culture were estimated. The share of shoots regenerating adventitious roots [%] was also determined.

### 4.6. Biochemical Array

Biochemical analysis was performed for shoots of 10 untreated in vitro-grown control plants and all bleeding hearts recovered in the experiments after 60 days of recovery culture. To extract chlorophyll, 80% (*v/v*) acetone solution (Chemia, Bydgoszcz, Poland) and 50 mg fresh tissue samples were used, following Arnon [41]. As for anthocyanins, the Harborne method [42] with 100 mg tissue samples and methanol containing 1% (*v/v*) of HCl (Chemia, Bydgoszcz, Poland) was employed. The solution mixture was analyzed for chlorophyll *a*, chlorophyll *b*, total chlorophyll *ct,* and anthocyanins content using a spectrophotometer (UV-VIS 1601-PC SHIMADZU, Kioto, Japan). Adequate solvents used to extract a given group of pigments were applied as control solutions. Mean absorbance was determined at the absorption maxima (645, 663, and 530 nm for chlorophyll *a*, *b*, and anthocyanins, respectively). The pigment concentration per gram of fresh matter of shoots was calculated using the algebraic method following Arnon [41] and Harborne [42]. 

### 4.7. Genetic Stability Evaluation

The genetic fidelity of LN-derived plants after 60 days of recovery culture was assessed using randomly amplified polymorphic DNA (RAPD), inter-simple sequence repeats (ISSR), and start codon target polymorphism (SCoT) markers. A total of 70 samples produced from cryopreservation treatments (i.e., 16 from the vitrification technique, 25 from droplet-vitrification, and 29 from encapsulation-vitrification; Table 4), and 10 untreated in vitro-grown controls (clones) were included in the analyses. Total genomic DNA was isolated from fresh tissues using a Genomic Mini AX Plant Kit (A&A Biotechnology, Gdynia, Poland), according to the manufacturer’s instruction. Isolated DNA was stored in TE buffer (10 mM TRIS, 1 mM EDTA, pH = 8) at 4 °C. DNA concentration and purity were monitored with the Quantus™ fluorometer (Promega, Madison, WI, USA). The quality was further checked on 1.0 g·L^−1^ agarose gel (Blirt, Gdańsk, Poland). All plastics and consumables were provided by GenoPlast Biochemicals, Rokocin, Poland.

A total of 18 primers (6 RAPD, 6 ISSR, and 6 SCoT; Genomed, Warsaw, Poland; Table 3) were used for the polymerase chain reaction (PCR). PCR was performed in a 25-µL reaction solution containing 2 mM MgCl_2_ in Reaction Buffer; 1 mM dNTP Solution Mix; 1 µM single primer; 0.05 U·µL^−1^ Taq DNA polymerase; 0.8 ng·µL^−1^ template DNA (20 ng); and sterile, double-distilled water to volume (2×PCR Master Mix Plus kit, A&A Biotechnology, Gdynia, Poland). DNA amplification was performed in a BioRad C1000 Touch thermal cycler (Bio-Rad, Hercules, CA, USA) using the following reaction: one cycle of 4 min at 94 °C for initial DNA denaturation; 40 cycles of 1 min at 94 °C for denaturation, 40 sec at 42/46 °C for annealing (for RAPD/ISSR, respectively), and 2 min at 72 °C for DNA extension. The last cycle was followed by a final extension step of 4 min at 72 °C. As for the ScoT analysis, the following profile was applied: one cycle of 4 min at 94 °C for initial DNA denaturation; 35 cycles of 1 min at 94 °C for denaturation, 50 sec at 44 °C for annealing, and 2 min at 72 °C for DNA extension. The last cycle was followed by a final extension step of 8 min at 72 °C. The amplified DNA fragments were separated on 1.5 g·L^−1^ agarose gel DN- and Rnase-free (Blirt, Gdańsk, Poland) in a TBE buffer (90 mM TRIS, 90 mM boric acidy, 2 mM EDTA, pH = 8.0) at 110 V for 90 min (Biometra P25, Jena, Germany), and detected by staining with 18 µl ethidium bromide at a concentration 10 mg∙mL^−1^ for 300 mL of gel. Gel images were recorded using a GelDoc XR+ Gel Photodocumentation System UV transilluminator with the Image Lab 4.1 software (Bio-Rad, Hercules, CA, USA). Molecular weights of the fragments were estimated using a 100–5,000 bp DNA molecular marker (GPB5000bp DNA Ladder, GenoPlast Biochemicals, Rokocin, Poland).

For every primer tested, the numbers of monomorphic, polymorphic (present in the electrophoretic profile of more than one individual), and specific/unique markers (present in the electrophoretic profile of a single individual) were counted.

### 4.8. Statistical Analysis 

The experimental design was completely randomized, consisting of seven PVS3 exposure times and +/− exposure to LN. Each experiment was repeated four times with 5 (non-LN-stored) or 10 (LN-stored) shoot tips for each treatment. A total of 1200 shoot tips were used.

After the normality transformation, the results were statistically analyzed by the analysis of variance (ANOVA) and Newman–Keuls tests (*p* ≤ 0.05) using the Statistica 12.0 (StatSoft, Warsaw, Poland) and ANALWAR-5.2-FR Excel add-in tools. Tables with results provide real numerical data, while alphabetical letters indicate homogenous groups based on the transformed data.

Measures of genetic uniformity among recovered individuals were determined using Jaccard’s similarity coefficient [43]. The XlStat 365 Excel add-in software (Addinsoft, Paris, France) was used to draw the dendrograms based on Agglomerative Hierarchical Clustering with Unweighted Pair Group Average Method (AHC UPGMA). Population groups were distinguished using the GenAlex 6.5 software [44], based on the analysis of molecular variance (AMOVA) estimates with the assumption that each cryopreservation technique (V, DV, EV) and untreated control plants (C) comprise four separate groups. 

## 5. Conclusions

This is the first report on the successful cryopreservation of *Lamprocapnos spectabilis*. Among the three techniques evaluated, encapsulation-vitrification was the most effective, while the vitrification technique was the least effective. The encapsulation-vitrification protocol was the most successful in securing the viability of the explants and in stimulating their morphogenetic response (shoot proliferation and root formation). This technique was also the only one that did not impose extra mutational load on the plants. Cryopreservation caused a significant decline in chlorophyll content, compared to the untreated control. It also affected the concentration of anthocyanins in plants. Nevertheless, the optimized encapsulation-vitrification protocol detailed in this study, based on 150 min dehydration with PVS3, can be recommended for the long-term storage of genetic resources of *L. spectabilis* ‘Gold Heart’. A further extension to other cultivars or even plant species also seems possible, with no or little risk of somaclonal variation occurrence.

## Figures and Tables

**Table 1:** Influence of three cryopreservation techniques on the recovery level [%], number, length [mm], and fresh weight [mg] of shoots produced from a single viable explant (+/− LN-stored), and their capability to form roots [%] after 60 days of recovery culture.

Dehydration Duration [min]	Plant Material				
	Non-LN-Stored		LN-Stored		
	Non-Encapsulated	Encapsulated	Vitrification	Droplet-Vitrification	Encapsulation-Vitrification
Recovery [%}					
control	100 a	85.0 bc	0.0 l	0.0 l	0.0 l
0 (20 min LS)	100 a	81.7 b–d	0.0 l	0.0 l	0.0 l
30	80.0 b–d	85.0 bc	6.4 kl	52.4 fg	0.0 l
60	87.5 b	52.5 fg	25.8 hi	44.8 fg	32.4 h
90	42.3 gh	57.9 e–g	14.8 jk	22.8 hi	50.0 fg
120	51.8 fg	70.8 b–e	8.9 kl	23.0 hi	45.8 fg
150	28.8 hi	68.8 c–f	11.4 jk	26.3 hi	73.1 b-e
180	12.5 jk	62.5 d–g	13.3 jk	26.2 hi	40.3 gh
No. of shoots per explant					
control	2.0 ± 0.4 bc	2.2 ± 0.2 bc	-	-	-
0 (20 min LS)	1.9 ± 0.1 bc	2.1 ± 0.3 bc	-	-	-
30	2.1 ± 0.5 bc	1.8 ± 0.1 bc	1.0 ± 0.0 c	1.6 ± 0.2 bc	-
60	1.8 ± 0.2 bc	1.4 ± 0.3 c	3.1 ± 0.4 bc	2.3 ± 0.3 bc	2.5 ± 0.4 bc
90	1.9 ± 0.2 bc	1.5 ± 0.2 c	2.0 ± 0.2 bc	1.3 ± 0.3 bc	2.1 ± 0.3 bc
120	1.9 ± 0.2 bc	1.3 ± 0.3 c	3.0 ± 0.0 bc	1.3 ± 0.3 bc	4.9 ± 0.8 a
150	1.6 ± 0.2 bc	1.4 ± 0.3 c	1.0 ± 0.0 c	1.5 ± 0.3 bc	3.9 ± 1.4 ab
180	1.0 ± 0.0 c	1.9 ± 0.4 bc	1.5 ± 0.5 c	2.0 ± 0.6 bc	2.2 ± 0.3 bc
Shoot length [mm]					
control	17.1 ± 3.8 a–d	25.0 ± 1.9 ab	-	-	-
0 (20 min LS)	16.7 ± 0.7 a–d	29.3 ± 2.9 a	-	-	-
30	26.2 ± 3.9 ab	25.3 ± 1.5 ab	29.5 ± 0.5 a	9.9 ± 2.3 cd	-
60	12.9 ± 2.8 b–d	17.4 ± 1.4 a–d	17.0 ± 3.0 a–d	11.6 ± 1.4 b–d	14.0 ± 1.9 b–d
90	15.8 ± 1.5 a–d	19.5 ± 4.2 a–d	14.0 ± 1.7 b–d	6.8 ± 1.6 d	15.1 ± 2.2 a–d
120	23.6 ± 5.9 a–c	19.6 ± 3.7 a–d	11.7 ± 1.0 b–d	7.3 ± 1.7 d	17.9 ± 1.9 a–d
150	18.1 ± 4.6 a–d	18.4 ± 3.0 a–d	5.8 ± 0.4 d	7.9 ± 1.2 d	16.6 ± 0.4 a–d
180	10.0 ± 2.0 cd	15.0 ± 3.0 a–d	9.2 ± 0.2 cd	8.5 ± 1.5 d	16.4 ± 0.8 a–d
Shoot weight [mg]					
control	198.3 ± 88.3 c	324.1 ± 80.3 c	-	-	-
0 (20 min LS)	87.8 ± 9.4 c	517.4 ± 97.8 bc	-	-	-
30	451.0 ± 103.9 bc	321.1 ± 56.3 c	2412.5 ± 387.5 a	49.9 ± 22.3 c	-
60	73.7 ± 13.2 c	160.7 ± 70.0 c	739.3 ± 191.5 bc	146.4 ± 35.8 c	172.3 ± 57.2 c
90	194.5 ± 53.5 c	215.3 ± 110.7 c	176.6 ± 35.3 c	100.2 ± 82.0 c	206.7 ± 19.9 c
120	443.2 ± 203.3 bc	258.2 ± 126.4 c	126.6 ± 30.7 c	60.1 ± 26.0 c	951.8 ± 335.7 b
150	162.6 ± 61.0 c	239.6 ± 96.9 c	114.3 ± 40.8 c	100.2 ± 36.0 c	633.0 ± 323.8 bc
180	41.4 ± 5.2 c	220.0 ± 150.2 c	121.2 ± 72.2 c	205.8 ± 70.3 c	174.0 ± 31.9 c
Rhizogenesis [%]					
control	0.0 c	35.0 a	-	-	-
0 (20 min LS)	0.0 c	33.7 a	-	-	-
30	31.2 ab	17.5 ac	0.0 c	20.8 ac	-
60	20.0 ac	0.0 c	10.4 c	0.0 c	29.2 ac
90	0.0 c	0.0 c	0.0 c	0.0 c	12.5 bc
120	8.3 c	10.0 c	0.0 c	0.0 c	41.2 a
150	0.0 c	0.0 c	0.0 c	0.0 c	30.2 ac
180	0.0 c	0.0 c	0.0 c	0.0 c	16.7 ac ^1^

^1^ Means ± SE in rows and columns marked with the same letter do not differ significantly at *p* ≤ 0.05 according to Newman–Keuls test; LS, loading solution.

**Table 2:** Influence of three cryopreservation techniques on the concentration of chlorophyll *a*, chlorophyll *b*, total chlorophyll *ct*, and anthocyanins in +/− LN-stored shoot fresh matter [mg·g^−1^] after 60 days of culture.

Dehydration Duration [min]	Plant Material				
	Non-LN-Stored		LN-Stored		
	Non-Encapsulated	Encapsulated	Vitrification	Droplet-Vitrification	Encapsulation-Vitrification
Chlorophyll *a* [mg·g^−1^]					
control	3.24 ± 0.46 a	1.86 ± 0.37 d	-	-	-
0 (20 min LS)	1.67 ± 0.04 de	0.88 ± 0.07 de	-	-	-
30	2.68 ± 0.47 b	0.52 ± 0.04 e	1.12 ± 0.38 de	0.43 ± 0.19 e	-
60	2.56 ± 0.22 c	0.43 ± 0.12 e	0.76 ± 0.07 de	0.57 ± 0.00 e	0.72 ± 0.14 e
90	1.41 ± 0.06 de	0.49 ± 0.18 e	1.21 ± 0.00 de	0.54 ± 0.18 e	0.70 ± 0.16 e
120	1.88 ± 0.23 d	0.57 ± 0.07 e	1.12 ± 0.40 de	0.82 ± 0.38 e	0.88 ± 0.17 e
150	0.51 ± 0.01 e	0.44 ± 0.09 e	0.75 ± 0.23 de	0.64 ± 0.27 e	0.84 ± 0.11 e
180	0.37 ± 0.01 e	0.65 ± 0.10 e	1.39 ± 0.17 de	0.72 ± 0.16 e	0.47 ± 0.04 e
Chlorophyll *b* [mg·g^−1^]					
control	1.21 ± 0.17 a	0.66 ± 0.13 bc	-	-	-
0 (20 min LS)	0.64 ± 0.03 b–d	0.20 ± 0.02 c–e	-	-	-
30	1.12 ± 0.13 a	0.09 ± 0.01 e	0.32 ± 0.08 de	0.25 ± 0.10 c–e	-
60	1.00 ± 0.07 a	0.09 ± 0.07 e	0.34 ± 0.02 de	0.31 ± 0.00 de	0.20 ± 0.04 c–e
90	0.53 ± 0.01 b–d	0.11 ± 0.06 e	0.46 ± 0.02 de	0.28 ± 0.08 de	0.36 ± 0.10 de
120	0.74 ± 0.07 b	0.14 ± 0.03 e	0.45 ± 0.19 de	0.49 ± 0.01 de	0.34 ± 0.10 de
150	0.22 ± 0.00 c–e	0.10 ± 0.04 e	0.36 ± 0.06 de	0.37 ± 0.16 de	0.16 ± 0.02 e
180	0.36 ± 0.04 de	0.13 ± 0.04 e	0.51 ± 0.14 de	0.36 ± 0.06 de	0.17 ± 0.04 de
Chlorophyll *ct* [mg·g^−1^]					
control	4.44 ± 0.62 a	2.52 ± 0.50 bc	-	-	-
0 (20 min LS)	2.31 ± 0.06 b–d	1.08 ± 0.09 b–e	-	-	-
30	3.79 ± 0.56 a	0.62 ± 0.05 de	1.45 ± 0.45 b–e	0.68 ± 0.29 de	-
60	3.57 ± 0.28 a	0.52 ± 0.19 e	1.10 ± 0.08 b–e	0.88 ± 0.00 de	0.93 ± 0.17 c–e
90	1.94 ± 0.08 b–e	0.60 ± 0.24 de	1.67 ± 0.02 b–e	0.82 ± 0.26 de	1.07 ± 0.24 b–e
120	2.61 ± 0.30 b	0.70 ± 0.11 de	1.57 ± 0.58 b–e	1.31 ± 0.03 b–e	1.22 ± 0.27 b–e
150	0.73 ± 0.01 de	0.54 ± 0.05 e	1.11 ± 0.29 b–e	1.02 ± 0.43 b–e	1.00 ± 0.11 b–e
180	0.73 ± 0.05 de	0.78 ± 0.13 de	1.90 ± 0.30 b–e	1.07 ± 0.22 b–e	0.63 ± 0.05 de
Anthocyanins [mg·g^−1^]					
control	5.38 ± 0.87 c–g	6.76 ± 1.73 b–e	-	-	-
0 (20 min LS)	4.28 ± 0.67 c–i	4.54 ± 0.46 c–i	-	-	-
30	6.16 ± 1.57 b–f	4.00 ± 0.19 d–i	9.01 ± 1.49 ab	10.73 ± 0.05 a	-
60	8.86 ± 1.33 ac	3.59 ± 0.61 d–i	5.71 ± 0.64 b–g	2.89 ± 0.25 e–i	4.57 ± 0.44 c–i
90	2.54 ± 1.08 f–i	5.43 ± 0.07 b–g	3.50 ± 0.71 e–i	3.83 ± 0.13 d–i	6.98 ± 0.36 b–e
120	4.92 ± 0.63 c–h	4.24 ± 0.36 c–i	3.50 ± 0.25 e–i	1.88 ± 0.03 g–i	7.66 ± 0.35 a–d
150	1.88 ± 0.02 g–i	4.57 ± 0.37 c–i	0.51 ± 0.03 i	2.04 ± 0.52 g–i	8.15 ± 0.75 a–c
180	n.a.	6.55 ± 1.43 b–f	0.99 ± 0.08 hi	1.65 ± 0.16 h–i	1.10 ± 0.15 hi
Chlorophyll *ct* to anthocyanins ratio					
control	0.82 ± 0.15 b	0.37 ± 0.03 b	-	-	-
0 (20 min LS)	0.54 ± 0.07 b	0.24 ± 0.04 b	-	-	-
30	0.61 ± 0.25 b	0.16 ± 0.01 b	0.16 ± 0.00 b	0.06 ± 0.02 b	-
60	0.40 ± 0.03 b	0.15 ± 0.06 b	0.21 ± 0.02 b	0.30 ± 0.00 b	0.20 ± 0.03 b
90	0.76 ± 0.36 b	0.11 ± 0.04 b	0.48 ± 0.09 b	0.21 ± 0.08 b	0.15 ± 0.20 b
120	0.53 ± 0.04 b	0.17 ± 0.01 b	0.45 ± 0.15 b	0.70 ± 0.04 b	0.16 ± 0.04 b
150	0.39 ± 0.00 b	0.12 ± 0.01 b	2.18 ± 0.78 a	0.50 ± 0.23 b	0.12 ± 0.02 b
180	n.a.	0.12 ± 0.04 b	1.92 ± 0.02 a	0.64 ± 0.03 b	0.57 ± 0.17 b ^1^

^1^ Means ± SE in rows and columns marked with the same letter do not differ significantly at *p* ≤ 0.05 according to Newman–Keuls test; LS, loading solution; n.a., not available.

**Table 3:** Characteristics of molecular products and number/share of plants with polymorphisms detected with RAPD, ISSR, and SCoT markers.

Primer Symbol	Primer Sequence 5′→3′	Marker Size [bp]		No. of Markers Per Sample				Total Poly. Markers [%]	No. and (%) of Plants with Polymorphism
		Min.	Max.	Total	Mono.	Poly.	Spec.		
**RAPD**									
**R-A**	GAC CGC TTG T	655	2954	5	5	0	0	0.0	0 (0.0)
**R-B**	GGA CTG GAG T	308	1505	6	6	0	0	0.0	0 (0.0)
**R-C**	GCT GCC TCA GG	500	1840	8	8	0	0	0.0	0 (0.0)
**R-D**	CAA TCG CCG T	36	1069	6	6	0	0	0.0	0 (0.0)
**R-E**	GGT GAC GCA G	151	1004	9	9	0	0	0.0	0 (0.0)
**R-F**	CCC AGT CAC T	96	1005	9	7	1	1	22.2	1 (1.2)
**∑**				43 (7.2)	41 (6.8)	1 (0.2)	1 (0.2)	(3.7)	1 (1.2)
**(mean from a single primer)**									
**ISSR**									
**I-A**	GAG GGT (GGA)_2_ TCT	200	2063	8	3	5	0	62.5	2 (2.5)
**I-B**	C (GAGA)_4_	723	1426	5	4	0	1	20.0	1 (1.2)
**I-C**	C (AGAG)_4_	-	-	-	-	-	-	-	-
**I-D**	(GACA)_4_	333	1366	7	5	2	0	28.6	2 (2.5)
**I-E**	(GA)_9_ T	-	-	-	-	-	-	-	-
**I-F**	GT (GAGA)_4_	-	-	-	-	-	-	-	-
**∑**				20 (6.7)	12 (4.0)	7 (2.3)	1 (0.3)	(37.0)	3 (3.8)
**(mean from a single primer)**									
**SCoT ^1^**									
**S-A**	CAA CA**A** **TG**G CTA CCA CCG	420	850	5	5	0	0	0.0	0 (0.0)
**S-B**	CAA CA**A TG**G CTA CCA CCT	310	3158	15	15	0	0	0.0	0 (0.0)
**S-C**	ACC **ATG** GCT ACC ACC GTC	325	880	8	8	0	0	0.0	0 (0.0)
**S-D**	ACC **ATG** GCT ACC ACC GTG	393	2290	8	8	0	0	0.0	0 (0.0)
**S-E**	CC**A TG**G CTA CCA CCG CCA	375	1182	7	7	0	0	0.0	0 (0.0)
**S-F**	CC**A TG**G CTA CCA CCG CAG	214	2320	8	8	0	0	0.0	0 (0.0)
**∑**				51 (8.5)	51 (8.5)	0 (0.0)	0 (0.0)	(0.0)	0 (0.0)
**(mean from a single primer)**									

^1^ RAPD, randomly amplified polymorphic DNA; ISSR, inter-simple sequence repeats; SCoT, start codon target polymorphism; mono., monomorphic; poly., polymorphic; spec., specific (unique; present in a single band profile); total poly., share of polymorphic and specific markers (together) in the total number of markers.

**Table 4:** Number of samples from cryopreservation treatments and untreated controls used in molecular analyses.

C	DV 30	DV 60	DV 90	DV 120	DV 150	DV 180	EV 60	EV 90	EV 120	EV 150	EV 180	V 30	V 60	V 90	V 120	V 150	V 180
10	3	7	3	3	5	4	7	5	7	5	5	2	4	3	2	3	2 ^1^

^1^ C, control; DV, droplet-vitrification; EV, encapsulation-vitrification; V, vitrification.

## Abbreviations

AHC	Agglomerative Hierarchical Clustering
AMOVA	Analysis of molecular variance
ANOVA	Analysis of variance
BA	6-benzyladenine
bp	Base pair
C	Control
DNA	Deoxyribonucleic acid
DV	Droplet-vitrification
EV	Encapsulation-vitrification
FW	Fresh weight
GA_3_	Gibberellic acid
ISSR	Inter-simple sequence repeats
LN	Liquid nitrogen
LS	Loading solution
Me_2_SO	Dimethyl sulfoxide
MS	Murashige and Skoog (1962)
PCR	Polymerase chain reaction
PLBs	Protocorm-like bodies
PPFD	Photosynthetic photon flux density
PVS	Plant vitrification solution
RAPD	Randomly amplified polymorphic DNA
SCoT	Start codon target polymorphism
SE	Standard error
TEM	Transmission
UPGMA	Unweighted Pair-Group Average Method
V	Vitrification
wm	Weight marker
WS	Washing solution
